# Archeo‐Inspiration from the Cultural History of Glass: Historic Accounts, Anecdotes and Hard Facts as Challenges to Modern Material Science

**DOI:** 10.1002/adma.202512937

**Published:** 2025-12-14

**Authors:** Eva von Contzen, Julia von Ditfurth, Fabian Stroth, Bastian E. Rapp

**Affiliations:** ^1^ Medieval English Literature Director of the Centre for Medieval Studies English Seminar University of Freiburg Rempartstr. 15 79085 Freiburg Germany; ^2^ Medieval Visual and Cultural Studies Institute of Art History University of Freiburg Platz der Universität 3 79085 Freiburg Germany; ^3^ Corpus Vitrearum Medii Aevi (Academy of Sciences and Literature Mainz) Institute of Art History Lugostraße 13 79100 Freiburg Germany; ^4^ Byzantine Archaeology Institute of Archaeological Studies (IAW) University of Freiburg Platz der Universität 3 79085 Freiburg Germany; ^5^ Laboratory of Process Technology NeptunLab Department of Microsystems Engineering (IMTEK) University of Freiburg Georges‐Köhler‐Allee 103 79110 Freiburg Germany; ^6^ Freiburg Materials Research Center (FMF) University of Freiburg Stefan‐Meier‐Straße 21 79104 Freiburg Germany; ^7^ FIT Freiburg Center of Interactive Materials and Bioinspired Technologies University of Freiburg Georges‐Köhler‐Allee 105 79110 Freiburg Germany

**Keywords:** archaeo‐inspired materials, archeology, Byzantium, functional historic materials, glass, historic materials, middle ages, nanoparticles, stained glass

## Abstract

Since its earliest developments, glass has held a unique place in cultural history. Remarkably, many surviving glass artefacts from premodern times still surpass some of today's standard processing techniques, with effects and qualities that feel strikingly contemporary. Valued for its optical purity, chemical and thermal resistance, hardness, and fragile beauty, glass has long captivated both artists and craftsmen. This paper proposes examining glasses within their cultural contexts as a source of inspiration for modern materials science. Throughout history, glass has held societal and symbolic significance, with diverse cultural accounts detailing its properties and uses. While not all historical records describe glass with scientific accuracy, many offer imaginative perspectives that can inform new developments. Just as fields like bionics and biomimetics look to nature for innovation, a culturally reflective scientific approach is suggested to glass material science, a concept “Archeo‐Inspiration” is termed. This concept draws from the material knowledge and creative uses of past societies to inspire future advancements in glass technology and material systems. The aim is to move beyond purely technical evaluation by reconnecting with the rich heritage of glassmaking within its cultural and historical framework. In doing so, the hope is to offer both a retrospective appreciation and a forward‐looking vision for material systems in the 21st century, grounded in the enduring legacy of one of humanity's most versatile and symbolically charged materials.

## Graphical abstract

Glass, historically valued for its purity and durability, has long inspired artists and societies. This article introduces the concept of “Archeo‐Inspiration”, drawing on cultural and historical contexts of glass to guide future material innovations. By revisiting ancient craftsmanship, modern science can find imaginative pathways for developing advanced glass technologies and material systems.

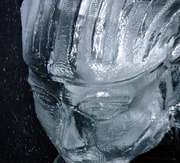

## 1 Introduction

The history of humankind has always been linked closely to materials. The ages, after all, are named after the most important material innovations. The *Bronze Age* represents one of the earliest periods in which advances in material technology, particularly metal alloying, led to profound societal changes, enabling new forms of craftsmanship, long‐distance trade, and social complexity. The *Iron Age* introduced harder, more durable tools and weapons that reshaped agriculture, warfare, and infrastructure across continents.^[^ ^1^ ^]^ The Roman innovation in the use of concrete marked a major material advancement with significant societal impact.^[^ ^2^ ^]^ Material innovation has had significant implications for society to a degree that one could argue material technology and society co‐construct each other.^[^ ^3^ ^]^ From the manufacturing of tools for improving the quality of life^[^ ^4^ ^]^ to warfare^[^ ^5^ ^]^, new materials and innovations in material processing have historically had a significant impact on society. The discovery and technological mastery of plastics has revolutionized consumer culture (with all its advantages and disadvantages)^[^ ^6^ ^]^, giving us access to one of the most important process technology innovations of the 21st century. Silicon processing has made semiconductor technology a mass‐market technology, enabled the microelectronics revolution, has put supercomputers at the tips of our fingers and fueled many of the most important technological breakthroughs of recent decades.^[^ ^7^ ^]^ Interestingly, many of the most recent material innovations build on a rich history of process technology, knowingly and unknowingly.

In this paper, we want to elucidate a technologically highly relevant high‐performance material: glass. Glass is one of the oldest artificial materials of humankind and has a history that is tightly interwoven with society and culture, and has held a unique place in cultural history.^[^ ^8^ ^]^

## 2 The Cultural Significance of Glass

Glass is one of the most important high‐performance materials of our times and is commonly associated positively as a material of value.^[^ ^9^ ^]^ In technical applications, glass is often chosen due to its outstanding chemical and thermal resistance as well as its optical properties.^[^ ^10^ ^]^ The duality of a mechanically strong yet brittle and fragile material has, over millennia, provided authors with a plethora of ideas to be transposed to literary and written contexts. Resulting from a complex manufacturing process, glass bears no resemblance to the starting material.

Human glass production can be traced back around 4,000 years and is thought to have originated in Mesopotamia.^[^ ^11^, ^12^ ^]^ Over time, glassmaking centers emerged across the Mediterranean. Glass beads, frequently found in excavations, rank among the earliest and most widespread glass artifacts. Early techniques for shaping glass included casting in open molds and core‐forming, in which a clay core was coated with molten glass and then removed to create hollow vessels. For many centuries, glass remained a luxury commodity, accessible primarily to the elite strata of society. This changed fundamentally with the most important innovation in glass history – the invention of glassblowing in the 1st century AD.^[^ ^13^ ^]^ It was only with the advent of the glass pipe that the usage of glass experienced a rapid expansion. This growth affected not only the volume of production but also the diversity of forms, sizes, and types of vessels and objects. These included common items such as cups, bottles, and lamps, as well as more specialized objects, like medical glassware and window glass (ref. [14], p. 104). While natron (sodium carbonate), in use as a flux since Roman times, remained common in Byzantium well into the Middle Ages for producing clear, stable glass of high optical quality, potash (potassium salts) gradually replaced it in the West from the 8th/9th centuries onward, resulting in the greenish *Waldglas* characteristic of northern Europe for centuries.^[^ ^15^ ^]^ A special feature of glass is, on the one hand, the dependence of the production process on certain raw materials and, on the other hand, on highly complex technical know‐how.

**Figure 1:**
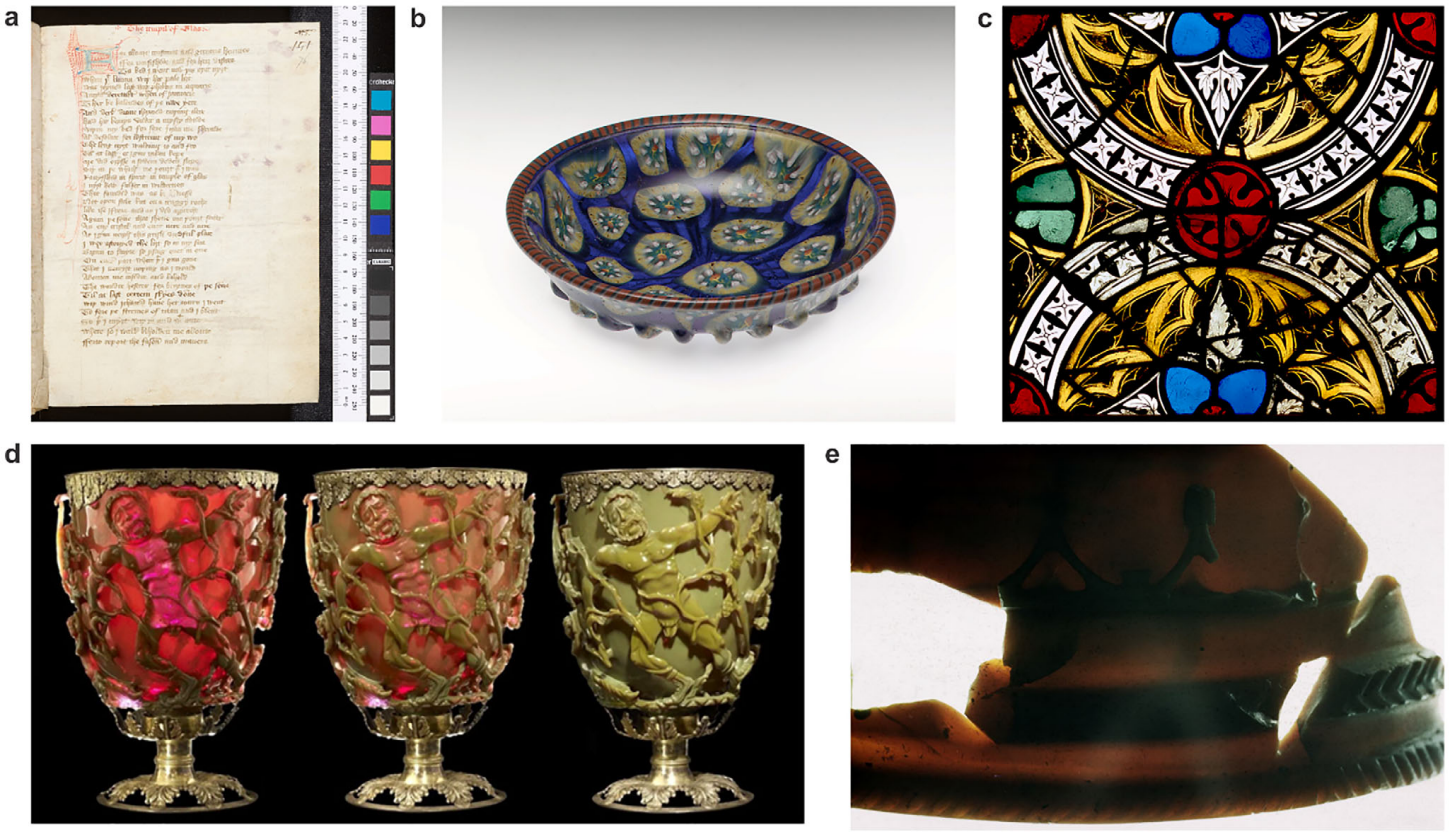
Examples of glass in historic contexts. a) Title page of Lydgate's “The Temple of Glass”, Bodleian Library MS Tanner 346, fol. 76r (mid‐15th century), Bodleian Libraries, University of Oxford (CC‐BY‐NC 4.0). b)  Bowl, Murano 19th c., MET (Inv. 25.78.147). This ribbed bowl was made using the ancient mosaic glass technique: fused murrine slices shaped in a mold. Each features a tulip‐like flower surrounded by seven tiny faces.; Image: public domain, c) Stained Glass Windows with tracery ornaments (ca. 1325/30), Freiburg, CVMA, previously located in Straßburg, St. Thomas; Image: Ulrich Engert, Corpus Vitrearum Deutschland/Freiburg i. Br. (CC BY‐NC 4.0). d) The Lycurgus Cup displays a remarkable color shift, appearing red when illuminated in transmission (left) and green in reflection (right). Although its dichroic properties have been recognized for centuries, the precise mechanisms behind this phenomenon were not understood until the work of Michael Faraday.^[^ ^20^, ^21^ ^]^ Image: The Trustees of the British Museum, object on display (G41/dc11)(G41/dc11), reprinted with permission. e) Cage Cup Situla from Termes, Museo Arqueológico Nacional Madrid, Inv. 21529; Image: RGZM in the 1960s, today Leibniz‐Zentrum für Archäologie (LEIZA).^[^ ^22^ ^]^

As the techniques of glass‐making evolved over time and new methods enabled glass‐makers to create more colorful and more intricate objects made of glass, glass also became an important material for critical reflection and poetic experimentation, especially in romances and dream visions.^[^ ^16^ ^]^ Geoffrey Chaucer's dream vision, *The House of Fame* (late 14th century), features an impressive temple made of glass, the temple of Venus, a building which the poet John Lydgate took up as the main setting in his *Temple of Glass* (early 15th century, see **Figure** **1**a). In both poems, the dreamer‐protagonists encounter buildings of astonishing beauty and brightness, which have painted images on their walls. In these poems, glass walls and stained‐glass windows become canvases for storytelling. They are mirror‐like in that they reflect older practices of narration and traditions. The texts thus invite comparison with the materiality of glass in order to express the affordances of text and writing.

In medieval philosophical and encyclopedic traditions, glass occupied a special position at the intersection of material reality and imaginative speculation. Texts such as Bartholomaeus Anglicus's *De proprietatibus rerum* and its Middle English translation by John Trevisa, drawing on Isidore of Seville's *Etymologiae*, combined technical, mythic, and geographical accounts of glass, depicting its origins in Phoenicia alongside fantastical descriptions of lands like Iceland and Gadis where glass possessed wondrous properties (ref. [17], p. 328). Beyond its physical qualities, glass held symbolic and theological significance: as the “vitreous humor” of the eye, it was linked to sight and cognition, while biblical and moral writings, notably Paul's “seeing through a glass, darkly” (1 Corinthians 13:12), framed it as a metaphor for spiritual imperfection and divine illumination.^[^ ^18^, ^19^ ^]^ This convergence of material, physiological, and metaphysical meanings made glass a central medium through which premodern thinkers explored perception, morality, and the relationship between the visible and the divine.

Taken together, these observations highlight that glass has always been more than a mere material: it is a site of knowledge transfer where craftsmanship, science, and imagination intersect. The historical trajectory of glassmaking, from ancient experimentation to medieval philosophical and poetic reflection, reveals how material understanding has long been shaped by cultural and intellectual frameworks. Recognizing this interplay opens productive pathways for contemporary and transdisciplinary research: historical sources do not simply document past practices but can actively inform and inspire present‐day innovation. By revisiting early techniques, symbolic associations, and conceptualizations of glass, modern materials science can draw on centuries of empirical insight and creative thinking to develop new approaches to design, functionality, and sustainability.

## 3 Archeo‐Inspiration: Historical Sources as Drivers for Modern Material Innovations

Material innovation does not happen at random. Innovation builds on decades, if not centuries, of improving skillsets, refined craftsmanship, and increases in our knowledge of the intricacies of a certain material. For every new generation, it may not be inherently obvious how much we are *standing on the shoulders of giants*, how much we rely on traditioned knowledge in material processing. We know from historical sources that specialized knowledge was often passed down from master to pupil and that many processes were kept as the historical equivalent of a trade secret. It is thus often difficult to judge if a certain process or technique is a technological innovation or a transfer of technique.^[^ ^23^ ^]^ A good example is the art of *Murano Glass‐*making (see Figure 1b), which was protected to the extent that master glassmakers were not allowed to leave the island of Murano out of fear that their knowledge would be passed on unintentionally or even via extortion.^[^ ^24^ ^]^ Unfortunately, this also implies that many process descriptions of how to make historically relevant materials do not allow us to directly replicate the process. Many of the methods for shaping glass were carefully guarded and transferred in close communication only. Although many glass specimens have been preserved across millennia, many of the most astonishing artefacts in glass have been lost. In order to accurately reconstruct the techniques that have been used to manufacture the most intricate glass objects in human history, it is essential to analyze written sources and surviving objects together and carefully evaluate the results. A good example of how difficult it is to replicate objects that were, at the time, very common is the example of stained glass windows (see Figure 1c). These are translucent works of art whose colors are unsurpassed in their luminosity, but whose manufacturing process cannot be easily discerned from the object itself. While statements about medieval wooden sculptures, for example, can be made on the basis of purely visual detection, because visible traces of processing on the surface provide information about the tools used, this is not possible for stained glass. Furthermore, there is the difficulty inherent in a medium that tends to be 2D, a difficulty that a 3D medium does not have. While the reproduction of 3D works of art, including architecture, in plaster, wood or bronze has been established in university teaching and museums since the 19th century, in the case of 2D works of art, such as panel painting, the preferred approach has been to use reconstructions in the form of pictorial representations, and replicas in the sense of casts or molds have not been tried. The number of surviving stained glass windows stands in sharp contrast to the paucity of surviving written sources. Some recipes and techniques have remained a mystery to this day. One can thus assume that many intricacies of glass making have been lost, and turning to historical sources and their cultural and literary contexts may reveal interesting aspects of glass making we may not be aware of today.

From the late 12th century onward, the development of Gothic architecture was accompanied by an increase in the size of window areas. The meter‐high, colored stained‐glass windows that now emerged became an essential characteristic of the Gothic style and brought colored light into the church interior, which was interpreted as transcendent, divine light.^[^ ^25^, ^26^ ^]^ The colored glass used for stained glass in the Middle Ages, the so‐called *Waldglas* or wood ash glass, consists of two‐thirds beech wood ash (potassium carbonate, (K_2_CO_3_), calcium carbonate (CaCO_3_), magnesium carbonate (MgCO_3_)), and one‐third river sand (silicon oxide, SiO_2_) (ref. [27], pp. 183–193). By heating it to a maximum of 750°C, a coherent glass mass is produced, the so‐called *frit*, which is heated further to around 1100°C and melted into the final glass mass before the cooling and molding processes. Metal oxides are decisive for the color of the glass, and these are naturally contained both in the beech wood ash (manganese oxides) and in the river sand (iron oxides) in proportions that vary depending on the location. A wide range of colors can be achieved with these, including metal oxides, and it was also possible to add cobalt for intense blue and copper for red glasses (ref. [28], pp. 13, 15–16). These colorants were partly added to the frit by adding glass pieces or mosaic tesserae (ref. [27], pp. 152, 194–196). Replicating recipes based on written accounts is impossible without access to the (close‐to) original materials. One of the richest historic sources on glass making is the second volume of the three‐volume *Schedula diversarum artium* (≈1100–1120) by the Benedictine monk Theophilus Presbyter, who provides a structured, detailed guide to glass production: from the construction of the glass furnace and the basic materials required to how to handle them and the repair of broken glass (ref. [27], pp. 143–165). The *Schedula* is the most important source on glass production in the High Middle Ages, and its importance for the study of artistic techniques in the medieval period can hardly be overestimated. Theophilus describes how the glass mass is produced from the above‐mentioned basic ingredients of ash and sand, which change color from saffron yellow to purple‐red depending on how long it is in the kiln. Theophilus's description corresponds with modern chemical analysis (ref. [29], pp. 26–29, 109–110). Precisely because the proportions of metal oxides in the ash and sand could not be determined in advance, the melting time was decisive for the color, which in turn was influenced by external factors such as temperature. The frit, therefore, had to be monitored closely and processed further as soon as the desired color was achieved. If the color changed from yellow to reddish yellow, Theophilus recommended either removing the reddish yellow, flesh‐colored glass mass and using it for the incarnation of the figures in stained glass windows or boiling it further for a light or dark purple. Then as now, the exact color tone that the glass will have after cooling cannot be determined in advance, which is why even the most renowned glassworks of our time, like Lamberts in Waldsassen (Germany), still allow for a certain tolerance in individual nuances. Theophilus had planned four further chapters for the other colors, which, however, have only survived in the table of contents: XII. *De coloribus, qui fiunt et cupro et plumbo et sale* ('On the colors made from copper, lead, and salt'), XIII *De viridi vitro* ('On green glass'), XIV *De vitro saphireo* ('On sapphire‐colored glass'), XV *De vitro quod vocatur gallicum* ('On what is called *Gallic glass*', i.e., red glass). The chapters themselves are not preserved in any of the surviving manuscripts, but researchers have attempted to close this gap by taking into account the explanations of the otherwise unknown author Heraclius in *De coloribus et artibus Romanorum* (books 1–2: 8th century, book 3 extension of the 12th century) (ref. [27], p. 191 and ref. [30], pp. 79–88). Just how difficult (not to say unsolvable) this can be is shown by the color blue and the *materia saphirorum* mentioned in Abbot Suger's writings on the stained glass in St. Denis in France, which still poses great mysteries and challenges to art historical research today: art historians disagree about what the term *materia saphirorum* actually means. Does it refer to a specific material (used to color glass?), and if so, either the transparent gemstone sapphire or the opaque lapis lazuli, or is it a metaphorical reference to the color of the earthly sky and thus, in turn, a reference to the divine? (ref. [31], pp. 78–95 and ref. [32], pp. 1–49). In other words, even if written sources exist that at first glance provide information about the material from which stained glass windows were made, on closer inspection, they may not deliver results for material science, but rather more discussion material for the humanities. It is natural to assume that these primary texts could yield rich inspiration for modern material science and, taken within their contexts, would help guide future research in the field. Interdisciplinary research on certain aspects of historic glass, such as, e.g., the aforementioned stained glasses, has been undertaken by art historians and restorers specializing in medieval stained glass at least since the Second World War, and the founding of the internationally associated *Corpus Vitrearum Medii Aevi* stained glass research centers (https://corpusvitrearum.org/).^[^ ^33^ ^]^ Archaeometric methods have been introduced,^[^ ^28^ ^]^ but material research has not (yet) had a strong focus in this work.

The idea of taking historical sources for inspiration on material processing has recently been coined “paleo‐inspiration”^[^ ^34^ ^]^ with a more general look into the material chemistry and composition. This approach is similar to “bio‐inspiration”, i.e., taking natural systems and structures as inspiration for technical systems. The term “historical bio‐inspiration” has been defined as taking old image and text sources on natural systems for inspiration on novel concepts.^[^ ^35^ ^]^ In analogy, we here define the concept of “archeo‐inspiration” as the reinterpretation of ancient or medieval techniques, with a focus not only on the material chemistry, but on the material systems, i.e., the interplay of material and process technology, which would allow to redesign either historically documented and (in several cases) still accessible artifacts but also to implement functions which have been described in historical sources only and which, to this date, have not (yet) been realized. In a sense, we would like to take historical knowledge as a basis and historical sources as a challenge for elucidating the potential future of glassmaking. We argue that a transdisciplinary, archeo‐inspired approach to material studies can lead to innovative insights for state‐of‐the‐art production techniques.

Many of the artefacts of glass that survive from premodern contexts today still surpass the predominant methods of glass processing in the 21st century, and many of the effects found in historical specimens have a surprising actuality. As an example, optical metamaterials have been documented over a span of two millennia, with current technology hard‐pressed to replicate the craftsmanship required to make these objects. Our aim is to explore the cultural and historic context of glass and the breakthrough developments which have, for various reasons, been largely ignored by modern developments in the material. One reason for this neglect is the modern division into specialist research fields: some areas of knowledge, especially historical knowledge, have been confined to disciplines such as archaeology, art history, or cultural studies, so that research into the history of materials has been carried out separately from research into the development of new materials. Bringing together the history of materials and their cultural and social traces with state‐of‐the‐art material research promises to foster a more holistic view of glass as a material. The evolution of glass craftsmanship, as well as the invention of novel processes, materials, and colors, has always been accompanied by a cultural echo of this material and thus, in the true sense of the word, a reflection of why the history of glass‐making has been such a success story.

## 4 Examples of Current Archeo‐Inspired Research Trends in Glass

### 4.1 Motivation

In the following, we will introduce three distinct research aims and trends which we would classify as areo‐inspired. They all take inspiration from historical context, and at least some of them have yielded very tangible innovations in glass.

### 4.2 Dichroic Glasses: Nanotechnology and 3D Printing

The first example we propose may be the most well‐known, as the artifact in question is one of the most precious pieces of human history. Achilleus Tatius's romance *Leucippe and Clitophon*, written at the end of the 2nd century AD, features the description of a precious glass drinking vessel. In the context of a festival of the god Dionysus, there is mention of a magnificent cup which has remarkable properties:

On that day, they celebrated the feast of the god. My father, in his eagerness for glory and to make the festival more splendid, also erected a krater (vessel) sacred to the god, which took second place to the krater of Glaucus of Chios. The whole work was made of glass of exquisite workmanship. It was entwined with vines that seemed to grow out of the crater. The grapes hung all around; each one appeared unripe when the crater was empty, but when wine was poured into it, it gradually began to change color and become a ripe grape. (Achilles ref. [36], p. 61)

At first glance, the mention of a drinking vessel that could change its color may seem unlikely, but there is in fact a glass of this type from the late ancient world: the *Lycurgus Cup* (ref. [37], pp. 119–121) (see Figure 1d). This late 4th‐century Roman cut glass vessel represents one of the outstanding achievements of the ancient glass industry.^[^ ^38^ ^]^ The piece is exceptional in a number of respects: first, in terms of the method of production and the exceptional workmanship involved, and second in terms of the unusual optical effects produced by the glass.^[^ ^39^, ^40^ ^]^ A mythological frieze depicting the legend of King Lycurgus from the sixth book of Homer's *Iliad* forms the openwork decoration of the Lycurgus Cup. The triumph of Dionysus over Lycurgus is depicted in deep relief. The glass of the cup is dichroic: In direct light (reflected light), it resembles jade with an opaque greenish–yellow hue, but when light passes through the glass (transmitted light), it changes to a translucent ruby red color. In a sense, this object may be the oldest object on record to show optical metamaterial properties, well over 1600 years before the inauguration of this terminology. The color change may have symbolized the way ripe grapes turn to wine, befitting the occasion of a Dionysian feast (ref. [37], p. 121). The nature of this effect has been studied in great detail, but given the delicacy of the object, many of the classical analytical techniques cannot be applied directly.^[^ ^41^, ^42^ ^]^ The distinctive effect is produced by gold and silver nanoparticles that nucleate within the glass as it cools from the melt. Although the high temperatures involved in this process are typically unfavorable for stable nucleation, the presence of numerous additional particulates within the glass likely enables Pickering stabilization, which helps maintain the stability of these nanoparticles. It was ultimately Michael Faraday who demonstrated that the vivid coloration arises from gold sols composed of extremely fine particles.^[^ ^20^, ^21^ ^]^

**Figure 2:**
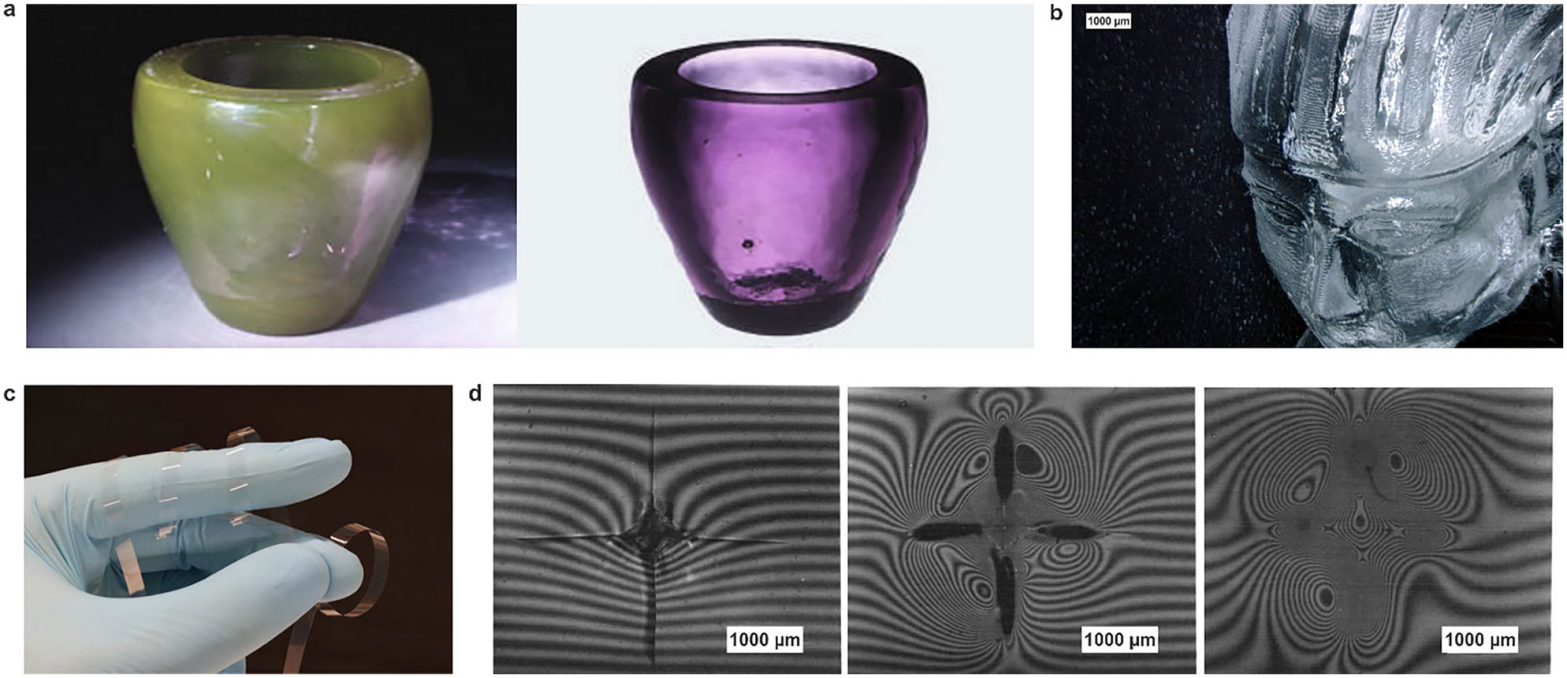
Examples of research trends in the field of glass material science with a strong connection to archeo‐inspired concepts. a) Example of dichroic glass as produced by Corning Glassworks (left: reflecting view; right: transmission view); image: adapted from [42], reprinted with permission. b) With the advent of high‐resolution additive manufacturing of glass using the Glassomer Technology,^[^ ^46^, ^48^ ^]^ complex glass components can now be manufactured and thus, structuring complex ornamental details, such as, e.g., required for the Lycurgus Cup, are now possible; Image: NeptunLab and Glassomer GmbH, reprinted with permissions. c) Ultrathin ribbons of a NEG silicate glass ribbon (thickness 20 µm), which can be bent flexibly; Image: adapted from ref. [50], reprinted with permission. d) Interferogram of a damaged glass surface and subsequent healing of the crack after 0 h (left), 1 h (middle), and 5 h (right) at elevated temperature. The initial crack starts healing as the glass reflows viscously, closing the gap. This process is sped up at higher humidity; Image: adapted from ref. [51], reprinted with permission.

Given the exceptional degree of intricacy of the ornamentation, it is astonishing, to say the least, that such an object could have been created almost two millennia ago. As discussed, it is very likely that the craftsmanship required to make dichroic glasses was passed on, as indicated by several other specimens exhibiting similar color effects^[^ ^22^, ^43^ ^]^, e.g., the *Cage Cup Situla* from Termes (see Figure 1e). While there have been successful attempts to replicate the dichroic nature of the cup material in glass^[^ ^44^ ^]^, the remaking of the intricate structure of the cup is very challenging. The Corning Museum of Glass reproduced a blank of a material with similar chemical composition and internal structure using a classical method for glass processing, showing the green/red color change effect under reflected and transmitted light^[^^]^ (see **Figure** **2**a). However, replicating the effect in glass is only the first step ‐ the (arguably) more challenging aspect is replicating the finesse of the structure. In contrast to other materials, most notable polymers, glass processing has seen little significant process innovation as the most common glass processing techniques on scale are still melt processing, grinding, and etching ‐ all of which have been used for centuries. The advent of high‐resolution 3D Printing of glass has been a joint effort by many members of the international glass community, which was recently compiled into the first state‐of‐the‐art technology overview.^[^ ^45^ ^]^ Our own group has contributed the Glassomer® Technology as a nanocomposite based approach to high‐resolution 3D Printing of silica glasses down to a resolution of a few micrometers which we first introduced for micro stereolithography^[^ ^46^ ^]^ as well as Two‐Photon Polymerization (2PP)^[^ ^47^ ^]^ and, more recently, for Volumetric Additive Manufacturing (VAM)^[^ ^48^ ^]^ (see Figure 2b). The process involves the compounding of an organic binder with glass nanoparticles to result in a nanocomposite that can effectively be shaped like a polymer. This material is accessible to many polymer processing technologies inducing injection molding^[^ ^49^ ^]^ as well as 3D Printing.^[^ ^46^, ^48^ ^]^ Structures with a level of fineness exceeding the Lycurgus cup have been created to this date, and, given a suitably formulated nanocomposite which mimics the dichromic effect of the cup, can be implemented, should allow for to generation of similar structures. This is thus a combined material processing technique involving the correct material to produce the dichroic effect and 3D Printing to provide the structure. With these techniques, a potential avenue to replicate this detail of craftsmanship has become available, and the community is actively investigating the possibility of replicating the Lycurgus Cup. With this, after nearly two millennia, highly detailed, dichroic glass components could return to use.

### 4.3 *Vitrum Flexile* ‐ The Elusive Malleable Glass

Glass as a material has always spurred the imagination. A particularly pervasive story concerned the (lost) invention of unbreakable glass (*vitrum flexile*). Isidore of Seville, in his famous encyclopedia, the *Etymologies* (7th century), provides us with the full account, which goes back to a passage in the ancient novel *Satyricon* by the Roman writer Petronius Arbiter.

They claim that under Tiberius Caesar, a certain craftsman devised a formula for glass so that it would be flexible and pliable. And when he was brought before Caesar, he presented a drinking bowl to him, and Caesar indignantly threw it to the floor. The craftsman picked the drinking bowl up from the floor, where it had been dented as a bronze vessel would be. Then he took a small hammer from his pocket and reshaped the drinking bowl. When he had done this, Caesar said to him, “Does anyone else know this method of making glass?” After the craftsman swore that no one else knew, Caesar ordered him beheaded, lest, if this skill became known, gold would be regarded as mud and the value of all metals would be reduced – and it is true that if glass vessels became unbreakable, they would be better than gold and silver.(ref. [17], p. 328)

While later medieval authors expressed their doubt about the truth value of the story, the desire to have a material that is like glass and at the same time malleable evidently had a strong appeal to people, past and present. Although scholars have coined malleable glass to be “vulgar fiction” due to the numerous unfounded claims of various artisans, scholars, and alchemists to have been successfully created this material,^[^ ^52^ ^]^ the idea of a glass that cannot break has inspired many innovations. German chemist Otto Röhm was one of the well‐known material scientists working on ways to make glass unbreakable. He eventually settled for a mineral glass‐based safety glass he termed *LUGLAS*, one of the earliest products of the Röhm & Haas company, and one which spurred significant interest in glasses that do not shatter upon impact. In 1936, he presented the newest attempt at a malleable and unbreakable glass in the form of *PLEXIGLAS*, one of the most well‐known tradenames for poly(methyl methacrylate) (PMMA). This transparent acrylic material won the *Grand Prix* and a gold medal for “arts and techniques” at the World Fair in 1937. (‘Transparent acrylic constructions before and after 1950–from the 1935 Opel Olympia to the 1972 Olympic roof’)^[^ ^53^ ^]^ Plexiglas has seen countless applications over its almost 100 years of use due to its wide range of advantageous properties as a technical thermoplast. However, the name *PLEXIGLAS* will always remain a reminder that it was originally developed as a surrogate for glass. Over the years, many engineering polymers have been developed with the aim of providing glass‐like properties while maintaining the structuring capabilities, impact resistance, and malleability of polymers.^[^ ^54^ ^]^ Since the first discovery of the polymerization of cycloolefins by Truett,^[^ ^55^ ^]^ Natta,^[^ ^56^ ^]^ and Sarttori,^[^ ^57^ ^]^ cycloolefin copolymers (COP) have been considered a viable alternative to glass in many applications. When research on Vitrimers, i.e., reversibly crosslinkable thermosets, yielded materials with outstanding mechanical properties (paralleling regular crosslinked thermosets) while maintaining the processability and malleability of thermoplasts, the materials were introduced as “Silica‐Like Malleable Materials”.^[^ ^58^ ^]^ It is obvious that the search for the malleable glass has not lost any of its appeal over centuries, nor has the hunt for unbreakable glass. When Steve Jobs first showcased the very first *iPhone* at the historic debut of the phone in 2007, the surface of the device (for Jobs switched out the phone several times during the show)^[^ ^59^ ^]^ was scratched. Jobs had been carrying the device in his pocket, together with his keys. Only months before the global release of the *iPhone*, Jobs’ team reached out to Corning for a specialty glass variant, which, at the time, was called *Chemcor* and was only mildly successful in the market. Corning rebranded the chemically surface‐hardened glass *Gorilla Glass*. The nature of the hardness is due to a chemical hardening of a silicate glass by ion exchange, whereby larger potassium ions are introduced into the surface of the glass, resulting in compressive strengthening.^[^ ^60^ ^]^ It was featured as the subtitle of Apple's press release, which stated that the *iPhone* “Now Features Durable Glass Top Surface”.^[^ ^60^ ^]^ Of course, as anyone who ever dropped a modern smartphone will know, having this glass not only surface‐hardened but also malleable would be a superb combination, as hardened glass is still very brittle and breaks easily.

Technical glasses usually only reach a fraction of their predicted intrinsic strength because of their brittleness.^[^ ^61^ ^]^ This is mainly due to the fact that defects act as points of stress concentration and thus, eventually, the breaking point of the material is reached. As glasses are amorphous, there are no grain boundaries to trap emerging cracks, which ultimately leads to the spontaneous breaking of the bulk. The probability of defects scales with the volume, and thus, low‐volume glass components can become surprisingly elastic.^[^ ^50^ ^]^ Ultrathin sheets of glass with thicknesses in the range of 10–100 µm can, if the defect density is low, be used as flexible substrates (see Figure 2c) for e.g., photonics applications.^[^ ^62^ ^]^ Glass nanowires have been reported to exceed 10 GPa of tensile strength.^[^ ^63^ ^]^ Recently, amorphous oxides of aluminum have been reported as an example of a ceramic equivalent to break‐resistant glass. Under the right processing conditions and in the absence of all bulk defects, alumina can undergo viscous creeping, allowing elongations of up to 100 % and full plastic deformation.^[^ ^64^ ^]^ This work has sparked renewed interest in similar mechanisms in other oxides, including glass.

### 4.4 Longevity in Glass: Toward Self‐Repair and Self‐Healing

The concept of material self‐healing is a closely related topic, which often emerges in historical sources as well. There are several accounts of materials that can, after being broken, be healed under various conditions. Although many of these accounts are fictitious, there are numerous stories of materials that had self‐healing capabilities, most notably the Roman *opus caementicium*. The Romans mixed dry quicklime with volcanic ash and water in an exothermic reaction, which forms large chunks of lime in the bulk of the material. Over time, as cracks and pores form in the concrete and water penetrates the material, the liquid dissolves calcium from the lumps, which, upon reaction with water and ambient carbon dioxide, forms carbonates that seal the voids.^[^ ^65^ ^]^ Interestingly, mending of glass is a technique which dates back to (at least) the Middle Ages and is described in detail, e.g., by Theophilus Presbyter in his second book on the glass making and shaping of the Middle Ages (ref. [27], pp. 183–193). The recipe essentially suggests that a broken glass can be repaired by carefully applying sieved ash (rich in sodium and potassium) dissolved in water into the fracture, which is to be left to dry in the sun. Afterward, blue or green glass (rich in ions which lower the melting point) should be ground on (silica‐rich) porphyry stone and applied to the mend before exposing the glass to heat. The high ion concentrations will create a very low‐melting glass, which effectively seals the break. Drastic reduction in glass transition temperatures upon addition of small amounts of ions and salts is common in silicate glasses.^[^ ^66^ ^]^ Theophilus ascertains that glasses mended using this protocol are *habebis ad quos usus volueris*, i.e., fit for every purpose, indicating that the repair will yield an (almost) pristine material. This is a realistic assessment as this protocol would, by all means, give a very solid and durable mending of the glass.

The concept of self‐healing is ubiquitous in nature and is the prerequisite for systemic longevity and, ultimately, for survival. These mechanisms are often provided by the intrinsic self‐healing capability of the biology of living systems, but have been explored extensively for artificial materials, including polymers and composite materials, some of which are either triggered and/or driven by stresses induced upon material failure.^[^ ^67^ ^]^ Several technical systems provide the ability to self‐heal by implementing sealing materials within the structure, which, upon breaking, are released to first close and ultimately seal the defect. Intrinsic self‐healing in polymers as a consequence of mechanical forces was reported even on very early polymer systems, such as vulcanized rubber, already in the 1980s.^[^ ^68^ ^]^ This is an example of mechanochemical self‐healing, a process that gives rise to the self‐healing properties of many polymers.

Self‐healing of glass is more challenging due to the low mobility in the amorphous structure of the material. However, amorphous glasses are viscous materials, and so, under the right conditions, self‐healing can be observed. This process is due to the disruption and reformation of bonds in the crack zone and is a classic example of mechanochemistry, which was studied as early as 1970.^[^ ^69^ ^]^ It was found that the crack healing propagates along four distinct phases: 1) exposure of the crack to moisture below the glass transition temperature; 2) formation of a gel layer in the crack zone; 3) closure of the crack resulting from stress relief; and 4) drying of the crack zone.^[^ ^70^ ^]^ Theoretical models for humidity levels from 0.01% to 100% have been provided, and today, we have a sound understanding of the closure mechanism, which is based on hydrogen bonding‐mediated linkages of surface‐adsorbed water.^[^ ^71^ ^]^ The bond energy is due to the surface siloxane bonds or cationic bridges and thus supports a similar mechanism as used in sol/gel chemistry, which allows forming glass objects by condensation from silicic acid, its salts, or alkoxides. At augmented temperatures, the ability to flow viscously enhances, and thus glass can self‐heal not unlike a fluid^[^ ^51^ ^]^ supported by capillary forces^[^ ^72^ ^]^ (see Figure 2d). Still, compared to polymeric systems and, obviously, to the living environment, self‐repair and longevity in glass have not been achieved to a notable extent. Today, safety glasses, including e.g., bullet‐proof glass, are still implemented mostly as a layer‐wise composite system where the glass provides the hardness and a polymer provides the toughness. Still, mechanisms such as, e.g., the condensation of silicic acid to form glass‐like materials, a process referred to as petrification, a well‐known processes for self‐sealing and are used in many commercial products, e.g., for sealing of leaking hydraulic equipment such as boilers. This, again, is a process which dates back to prehistoric architectures and has been used since, at least, the Iron Age.^[^ ^73^ ^]^ Entrapping material for self‐sealing in bulk materials for mitigating fracture damage is a well‐known principle in many synthetic polymers and composites.^[^ ^74^ ^]^ Given the short timeline to catastrophic failure in glasses, similar mechanisms are not straightforward to implement in amorphous glasses, unless crack propagation can be slowed down. There are methods to achieve this, but these have yet to be explored for similar applications.

## 5 Conclusion

Historical accounts of materials are often clouded in rich and suggestive narratives that attribute properties to materials which may seem, from the mere material scientific standpoint, more fantasy than reality. This is true for glass in particular, as it has always inspired artists and craftsmen alike with its unique combination of properties: optically pure, chemically and thermally resistive, hard yet so fragile. Glass has, without a doubt, always held a very special place in cultural history. In this paper, we reasoned that much can be gained from examining historical accounts of glass and glasses within their cultural contexts, which can serve as rich sources of inspiration for modern materials science. While it is true that historical records are often vague and the properties of glasses are often exaggerated or underspecified, there is a surprising amount of scientific truth behind these accounts. Fields like bionics and biomimetics use bio‐mimetics or bio‐inspiration to find innovation in nature's solutions. We believe that using a culturally reflective scientific approach to glass material science, an approach we introduced as “Archeo‐Inspiration,” can help guide future advancements in glass technology. Examining three examples and several historic accounts of surprisingly advanced material process knowledge, we believe that beyond the mere technical evaluation, it is well‐worth to reconnect with the rich heritage of glassmaking in its cultural and historical framework which will offer not only an appreciation for artisans and craftsmen of the past, but can provide us with a forward‐looking vision for the future development of material systems in the 21st century.

With the recent breakthroughs in high‐resolution 3D Printing of glass, one of the key components in the manufacturing of delicate glass items has been achieved, i.e., the high feature resolution. Improvements in material chemistry have afforded new pathways toward colored and dichroic glass, and it is very likely that, before long, a reinterpretation of the Lycurgus Cup and similar delicate glass objects will be feasible. The recent breakthroughs in understanding crystalline material plasticity are promising research directions toward plastically deformable glasses, and although self‐healing in glasses still requires rather harsh conditions, there is relevant research in this direction. Which other research directions and material innovations could be imagined for glass? Based on our outline of Archeo‐Inspiration, several potential avenues can be foreseen. Historically‐inspired formulations of inorganic glass colors could be used as highly‐absorbing and strongly‐coloring coatings on glass panes, thus, potentially, allowing for printing an arbitrary image onto a 3D printed glass preform, which would, effectively, allow printing a stained‐glass window or window segment. Segmented glasses based on lightly crosslinked polymeric glassy composites could be used to mimic plasticity, thus enabling the evasive *vitrum flexile*. Using composite metallic/glassy nanocomposites could allow many of the descriptive reflections and color impressions of glasses to be engineered, paving the way to glasses that change colors upon an external stimulus. Inspiration can be a basis of innovation. In glass, many of the historic accounts of glass properties are fictional ‐ but engineering properties approaching them can be a worthwhile effort toward material innovation.

## Conflict of Interest

The authors declare no conflict of interest.
